# Filtering respiratory motion artifact from resting state fMRI data in infant and toddler populations

**DOI:** 10.1016/j.neuroimage.2021.118838

**Published:** 2022-02-15

**Authors:** Sydney Kaplan, Dominique Meyer, Oscar Miranda-Dominguez, Anders Perrone, Eric Earl, Dimitrios Alexopoulos, Deanna M. Barch, Trevor K.M. Day, Joseph Dust, Adam T. Eggebrecht, Eric Feczko, Omid Kardan, Jeanette K. Kenley, Cynthia E. Rogers, Muriah D. Wheelock, Essa Yacoub, Monica Rosenberg, Jed T. Elison, Damien A. Fair, Christopher D. Smyser

**Affiliations:** a Department of Neurology, Washington University School of Medicine, St. Louis, MO, USA; b Department of Radiology, Washington University School of Medicine, St. Louis, MO, USA; c Department of Pediatrics, Washington University School of Medicine, St. Louis, MO, USA; d Department of Psychiatry, Washington University School of Medicine, St. Louis, MO, USA; e Department of Psychological and Brain Sciences, Washington University School of Medicine, St. Louis, MO, USA; f Institute of Child Development, University of Minnesota, Minneapolis, MN, USA; g Department of Pediatrics, University of Minnesota, Minneapolis, MN, USA; h Masonic Institute for the Developing Brain, University of Minnesota, Minneapolis, MN, USA; i Center for Magnetic Resonance Research and Department of Radiology, University of Minnesota, Minneapolis, MN, USA; j Department of Psychiatry, Oregon Health and Science University, Portland, OR, USA; k Department of Psychology, University of Chicago, Chicago, IL, USA

**Keywords:** Resting-state fMRI, Respiratory filtering, Neurodevelopment, Neuroimaging, infant

## Abstract

The importance of motion correction when processing resting state functional magnetic resonance imaging (rs-fMRI) data is well-established in adult cohorts. This includes adjustments based on self-limited, large amplitude subject head motion, as well as factitious rhythmic motion induced by respiration. In adults, such respiration artifact can be effectively removed by applying a notch filter to the motion trace, resulting in higher amounts of data retained after frame censoring (e.g., “scrubbing”) and more reliable correlation values. Due to the unique physiological and behavioral characteristics of infants and toddlers, rs-fMRI processing pipelines, including methods to identify and remove colored noise due to subject motion, must be appropriately modified to accurately reflect true neuronal signal. These younger cohorts are characterized by higher respiration rates and lower-amplitude head movements than adults; thus, the presence and significance of comparable respiratory artifact and the subsequent necessity of applying similar techniques remain unknown. Herein, we identify and characterize the consistent presence of respiratory artifact in rs-fMRI data collected during natural sleep in infants and toddlers across two independent cohorts (aged 8–24 months) analyzed using different pipelines. We further demonstrate how removing this artifact using an age-specific notch filter allows for both improved data quality and data retention in measured results. Importantly, this work reveals the critical need to identify and address respiratory-driven head motion in fMRI data acquired in young populations through the use of age-specific motion filters as a mechanism to optimize the accuracy of measured results in this population.

## 1 Introduction

Over the last decade, utilization of resting-state functional magnetic resonance imaging (rs-fMRI) to study infant and toddler cohorts has shown increasing promise for understanding trajectories of typical and aberrant functional brain development (Azhari et al., 2020; Graham et al., 2015; Zhang et al., 2019; Eyre et al., 2021; Lin et al., 2008.). In this population, measures of spontaneous, infra-slow (<0.1 Hz) fluctuations in the blood oxygen level-dependent (BOLD) signal have been analyzed to investigate the earliest forms of resting-state networks (RSNs) and provide insight into the functional architecture of the developing brain (Eyre et al., 2021; Eggebrecht et al., 2017; Smyser et al., 2016; Smyser 2016; Toulmin et al., 2015; Doria et al., 2010; Smyser et al., 2010; Gao et al., 2009; Fransson et al., 2007). Understanding relationships within and across these networks holds great promise for characterizing typical functional brain development (Grayson and Fair, 2017), as well as the deleterious effects of neurological and neurodevelopmental disorders, including epilepsy (Liu et al., 2009), autism (Redcay et al., 2013; Feczko et al., 2018; Ray et al., 2014), and attention deficit hyperactivity disorder (Posner et al., 2014; Mills et al., 2018; Cary et al., 2017).

Beginning with Biswal's seminal report (Biswal et al., 1995), adult rs-fMRI processing pipelines have undergone substantive methodological development focused on refinement and optimization (Biswal et al., 2010; Lee et al., 2013; Luna et al., 2010; Mather et al., 2013; Matthews et al., 2006; Uddin et al., 2010), resulting in highly sophisticated modeling techniques and analysis methods. Across these approaches, basic preprocessing involves steps including correction for slice-acquisition time delays and intensity differences, regression of head motion and tissue nuisance regressors, spatial smoothing, low-pass filtering to remove non-neuronal signal, and atlas registration (Lee et al., 2013). Of these elements, approaches for accurately and effectively identifying and correcting for the effects of subject motion remain an area of ongoing investigation, and its importance for rs-fMRI analyses has now been well-established (Power et al., 2012; Satterthwaite et al., 2012; Van Dijk et al., 2012). Critically, subject motion negatively impacts functional connectivity measurements, both by decreasing the signal-to-noise ratio (SNR) and by biasing connection strength relative to the physical distance of connections (Ciric et al., 2017). The associated downstream effects can result in inaccurate interpretations of rs-fMRI results and brain-behavior relationships.

Recent advances in MRI scanning acquisition methods, namely simultaneous multi-slice (SMS) imaging have revealed respiration as another source of problematic motion in both multiband (MB) and single-band adult and adolescent datasets (Fair et al., 2020; Gratton et al., 2020; Feinberg and Yacoub, 2012; Moeller et al., 2010; Uğurbil et al., 2013; Xu et al., 2013). While current motion correction techniques target the removal of all motion artifact, they fail to distinguish perturbations due to respiration that, unlike spontaneous isolated head movements, do not result in BOLD signal disruption. Consequently, frame motion estimates targeted for removal may include a residual respiratory component that should be considered independently, often resulting in unnecessarily reduced data retention (Power et al., 2013). In recent studies, Fair et al., and Gratton et al., developed a technique to identify and correct for respiratory motion in BOLD data using a band-stop filter which adequately corrects for respiratory-induced magnetic field perturbations. In adults and adolescents, analyzing the framewise displacement (FD; a metric measuring head motion distance from frame-to-frame) trace of MB data reveals a high-intensity frequency component in the phase encoding direction of data collection indicative of respiratory artifact. Once identified, this spurious head motion can be removed by applying a notch filter selected based upon the frequency of respiration. This method yields a more representative motion trace, allowing for increased frame retention by targeting only perturbations driven by isolated, spontaneous head movements for final frame removal (Fair et al., 2020). As a result, the remaining BOLD data contain less noise and are of higher quality.

Infant and toddler rs-fMRI processing pipelines provide unique challenges and must effectively account for several technical factors due to the rapidly changing environment of the developing brain (Turesky et al., 2021; Raschle et al., 2012). Key differences include variations in the size, shape, and hemodynamic response of the brain, as well as unique motion and sleep patterns (Cusack et al., 2018). Movement is of particular interest because, unlike in adult studies, it is typically impractical to regulate the movement of young children during data collection, especially during natural sleep. Motion patterns in sleeping infants and toddlers differ from those observed in alert adults, often limiting the ability to acquire large quantities of low motion rs-fMRI data. Thus, it is important to understand and develop motion correction pipelines that address these unique motion patterns inherent to this population. It was previously uncommon to correct for respiratory motion in infant and toddler processing pipelines. This was in part due to the fact that younger individuals (from 6 months to 11 years) breathe at faster rates than adults (20–30 bpm versus 12–18 bpm) (Kliegman and Nelson, 2011), and until the arrival of SMS imaging, these high frequency changes were aliased to a lower frequency making them indiscernible in head motion estimates (Fair et al., 2020). Further, due to differences in body size and habitus, these subjects’ head movements are characterized by lower amplitude head and trunk displacement during respiration, which were assumed to have little to no effect on the B0 field of the scanner. For these reasons, the necessity and utility of a filtering approach for respiratory artifact similar to that employed by Fair and colleagues in adults and adolescents remains unexplored in this age range.

In this report, we demonstrate the effects of respiratory-driven movement artifact in infant and toddler rs-fMRI data across two independent cohorts analyzed using independent analysis pipelines. We first confirm the presence of respiratory artifact in both cohorts, illustrating its comparable effects as an important source of colored noise in data from both groups. We then demonstrate respiratory artifact-driven filtering of the FD trace as a critical mechanism to improve rs-fMRI data retention and quality. Finally, we test the utility of customizing filters by age group to achieve optimal data quality across toddler rs-fMRI studies.

## 2 Materials and methods

### 2.1 Data Collection

#### 2.1.1 Baby Connectome project (BCP)

MRI data from 141 scanning sessions collected from 96 infants and toddlers aged 8–24 months (age = 14.3 ± 4.2 months, female *N* = 46, white *N* = 75) as part of the Baby Connectome Project (BCP; Howell et al., 2019) were used in these analyses. The BCP study was approved by the University of Minnesota and University of North Carolina Institutional Review Boards and informed consent was acquired from the parents of all participants. This project aims to understand brain development through structural and functional connectivity during the first 5 years of life.

Participants were scanned on a Siemens 3T Prisma scanner with a 32-channel head coil. T1-weighted (TR = 2400 ms, TE = 2.22 ms, 0.8 mm isotropic), T2-weighted (TR = 3200 ms, TE = 563 ms, 0.8 mm isotropic), spin echo fieldmaps (SEFM) (TR = 8000 ms, TE=66 ms, 2 mm isotropic, MB=1), and rs-fMRI data were collected. rs-fMRI data (TR = 800 ms, TE = 37 ms, 2 mm isotropic, MB=8) were collected in both anterior→posterior (AP) and posterior→anterior (PA) phase encoding directions. Each BOLD run consisted of 420 frames (5.6 min) with a maximum of 4 runs (22.4 min) collected per scanning session. A subset of early scans (*N* = 60) were collected with a TR = 720 ms; all analyses were performed with TRs separated and combined. All scans were performed during natural sleep without the use of sedating medications.

#### 2.1.2 Early life adversity, biological embedding (eLABE)

A total of 36 24-month-old toddlers (age = 25.9 ± 2.8, female *N* = 15, white *N* = 7) from the Early Life Adversity, Biological Embedding (eLABE) study were used in this analysis. This study was approved by the Washington University Institutional Review Board. Informed consent was obtained from the parents of all participants. This project explores the relationship between maternal experiences during pregnancy and brain and neurodevelopment outcomes during early childhood. Participants were scanned on a Siemens 3T Prisma with a 64-channel head coil using an identical acquisition protocol to the BCP cohort (including BOLD TR = 800 ms). BOLD runs were collected in the AP direction with a maximum of 8 runs (44.8 min) per scanning session. As in the BCP cohort, all scans were performed during natural sleep without the use of sedating medications.

### 2.2 Data analysis

#### 2.2.1 fMRI analysis

##### 2.2.1.1 Functional data pre-processing - BCP

Data processing steps from the BCP cohort resembled that of the Adolescent Brain Cognitive Development (ABCD) cohort as described in Feczko et al., 2021. The structural T1-weighted image is processed through FreeSurfer (version 6.0; Fischl, 2012), providing a brainmask. Additionally, the T2-weighted image was used to better inform FreeSurfer segmentations. This refined brainmask was registered to an MNI template using the ANTs compressible fluid deformation algorithm (Fonov et al., 2011). Using this transformation, the rs-fMRI timecourses were also registered to the MNI template. Standard preprocessing steps were first performed beginning with demeaning/detrending across time. Next, denoising is performed using a general linear model. Denoising regressors include signal and motion variables. Signal regressors include mean timeseries, white matter, cerebrospinal fluid (CSF), and global signal based off FreeSurfer segmentations. Motion regressors include volume-based translational and rotational components and their 24P Volterra expansion (Friston et al., 1996). Bandpass filtering was then performed using a second order Butterworth filter in the range of 0.008 to 0.09 Hz.

Motion correction then was performed for both standard preprocessing and for downstream construction of the parcellated timeseries. FD was defined as the sum of absolute values of the differences in motion estimates between each frame. Frames were censored during demeaning/detrending if their FD value exceeded 0.2 mm. Consequently, the denoised beta values only included the remaining low motion frames. To avoid aliasing caused by missing timepoints during the bandpass filtering step of preprocessing, interpolation is used to replace missing frames and residuals are pulled from the denoising general linear model. Further, when extracting timeseries data for analysis, only data with FD less than 0.2 mm was used. *T1*-weighted, *T2*-weighted, and BOLD images were visually inspected for quality by experienced raters (at least 2 independent raters per image). Sessions with less than 75% aggregated passing rate on either anatomical or functional images were excluded.

#### 2.2.2 Functional data pre-processing - eLABE

Data were processed through a standard toddler EPI (BOLD) preprocessing pipeline using the 4dfp tool suite (ftp://imaging.wustl.edu/pub/raichlab/4dfp_tools/; Shulman et al., 2010) to remove non-neuronal variance. Functional data were first slice timing corrected and debanded to correct for asynchronous slice time shifts and systemic interleaved intensity differences between even and odd slice acquisitions according to the methods described in Power et al., 2012. Inter-volume motion was corrected using 4dfp's cross_realiagn3d_4dfp, and bias field correction was performed using FSL tools (Jenkinson et al., 2012). Additionally, readout distortion correction was estimated using FSL's topup on each participant's individual SEFM, and applied to the BOLD data using applytopup. Movement analysis was performed using rigid body motion correction to correct the timeseries for head motion within runs. *T1*-weighted MRI images were registered and resampled to an age-specific atlas target. This volumetric timeseries was then registered to a representative adult atlas target in Talairach space (711–2B). The first frame of the rs-fMRI timeseries was registered to this corrected *T1*-weighted image through affine transformation and combined with the transformation to atlas space to result in a timeseries of 3 mm isotropic voxels. Normalization was performed to scale the whole brain mode intensity to 1000 using one constant for each rs-fMRI run (Smyser et al., 2010). The registration of the *T1*-weighted image, atlas target, and rs-fMRI timeseries were manually inspected by experienced raters to ensure accuracy of individual processing result.

Additional standard functional processing steps were performed. Frame censoring was performed, where only data with FD less than 0.2 mm were used. Nuisance waveforms were regressed out of the timeseries including retrospective motion correction from the 24-Friston parameters, gray matter global signal, and regions of non-interest and their first derivative (white matter, ventricular, and extra-axial CSF). The data were then bandpass filtered in the range of 0.005 to 0.1 Hz to eliminate non-BOLD frequencies, and spatially smoothed.

#### 2.2.3 Calculation of framewise displacement (FD) trace

Movement analysis performed during rigid body motion correction allowed for tracking of head position and motion across all volumes across both data sets. This involved a 6-parameter transformation, tracking translational displacements along the X, Y, and Z axes, and rotational displacements across these three planes (pitch, yaw, and roll). Instantaneous frame displacement was defined as the sum of absolute values of the differences between frame-to-frame changes of these 6 parameters.

#### 2.2.4 Power Spectra of head motion estimates

In order to determine if the respiratory-driven head motion artifact was present in this age group, we used the approach described in Fair, et al., to visualize the time traces of the movement parameters in the frequency domain to reveal high intensity frequency artifacts indicative of respiration rate. The individual subject frequency spectra were analyzed to inform the central peak and notch size for band-stop filters under consideration. Specifically, the median high intensity component for each subject was used to inform the group cutoff frequencies. For the BCP data, this analysis was completed for sessions with TR = 800 ms and TR = 720 ms separately (see Supplemental Information (SI) “BCP TR Replication” SI Fig. 1). Filtering was applied in Matlab using the iirnotch (BCP) and butter (eLABE) functions based on institutional availability.

**Fig. 1:**
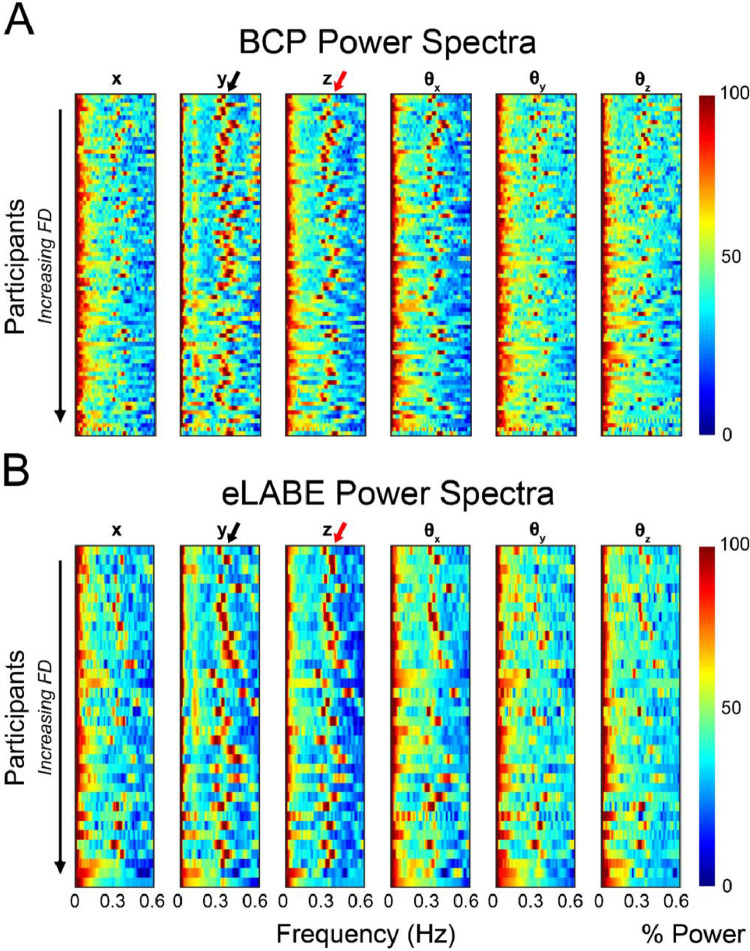
**Frequency domain representations of multiband rs-fMRI timeseries data.** (A) Power spectra for scans with TR = 800 ms (*N* = 81 sessions) from the BCP cohort and (B) 36 subjects from the eLABE cohort. Subjects are ordered by mean FD, with the lowest motion subjects organized at the top. Frequency transforms are computed across all directions for motion analysis, namely translation (X, Y and Z) and rotation (pitch, yaw, and roll). The red elevated power band between 0.3 and 0.5 Hz (indicated by the black arrow) in the phase encoding direction (Y) is evidence of respiratory artifact. The power spike in the non-phase encoding direction at this frequency (indicated by the red arrow) is likely a combination of true head motion and respiratory motion leak. High power motion artifact is suppressed in higher motion subjects, indicated by the gradual decline in the red high power band moving vertically down this plot.

#### 2.2.5 Determining Filter cutoffs

To determine the appropriate notch filter cutoffs for each cohort, first the individual median high intensity components were identified. From this, the median value across participants determined the central cutoff frequency and the second and third quartiles set the bandwidth for each cohort independently. Given the stability of respiratory rate from 1 to 11 years (Kliegman and Nelson, 2011), frequency cutoffs for the BCP cohort were calculated by combining all ages.

#### 2.2.6 Evaluating functional connectivity (FC) estimates

To obtain the fc values for regions throughout the brain, a pairwise correlation between the average BOLD timeseries within a standardized set of 333 cortical parcels (Gordon et al., 2016) was performed. These values were arranged into a connectivity matrix based upon age-specific RSN assignments that were determined using previously published methods (Wheelock et al., 2019; Eggebrecht et al., 2017). Briefly, the pairwise fc data from the 94 BCP subjects were averaged, producing a single 333×333 correlation matrix. The averaged connectome across BCP subjects was thresholded and binarized across a range of edge density thresholds (1% to 10% sparsity) and ROIs were assigned to functional modules using the Infomap community detection algorithm (Rosvall and Bergstrom, 2008). To obtain a general measure of RSN connectivity, the average correlation value of all parcels within and/or between networks was calculated. A connectivity matrix was created for each RSN using timeseries data from both before and after application of the respiratory notch filter. A paired *t*-test was performed for both the full connectivity matrix at the parcel level, as well as for each network average cell in the matrix to quantify improvement of fc estimate magnitude after application of the respiratory filter.

To further assess the effect of filtered FD on functional connectivity, a split-half protocol was implemented. Here, the BOLD runs for an individual subject were split into the first and second half of the total usable data defined at each FD threshold, thus each matrix consisted of equal amounts of data. For the BCP data, each half was made up of one AP and one PA run. Connectivity matrices were calculated for each half, and spatially correlated with one another across various FD thresholds. This approach was repeated for data with and without application of the respiratory FD filter to demonstrate how filtering increases or decreases split-half reliability across BOLD runs.

## 3 Results

### 3.1 Presence of respiratory artifact in infants and toddlers

Power spectra plots demonstrating the frequency representation of the head motion estimates are presented in Fig. 1 for both cohorts. In these results, a spike in power at the respiratory rate of toddlers (between 0.2 and 0.6 Hz) would indicate that the artifact is present in the data. The red band at this rate in both the BCP (Fig. 1a) and eLABE (Fig. 1b) data with TR=800 ms, indicated by the black arrow in the *y* direction plot, shows that the artifact is consistently present in data collected in this population. Note, the power also spikes in the non-phase encoding directions at this frequency, indicated by a red arrow in the z direction, which is likely respiratory motion leak (see Discussion).

### 3.2 Filtering FD trace to remove respiratory artifact

**Fig. 2:**
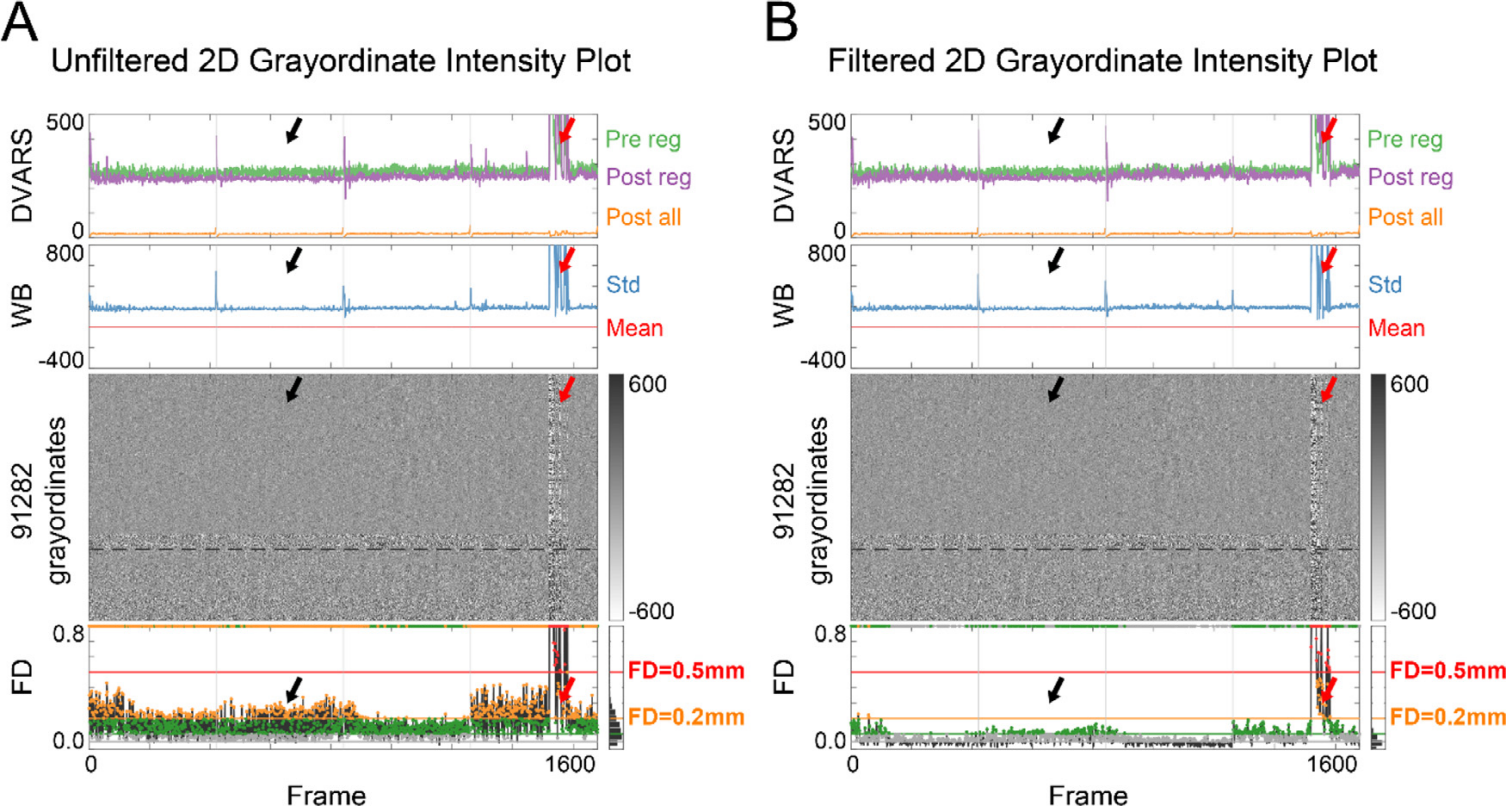
**Grayordinate intensity plot for an individual subject.** (A) Data are shown before and (B) after the application of a notch filter in the range of respiration. Grayordinate representation for a single subject tracking the spatial root mean square of the derivative of the timeseries (DVARS), the whole brain signal, the FD value and finally the grayordinate plot across the duration of the acquisition. With an FD threshold of 0.2 mm, indicated by the horizontal orange line, any frames that surpass this minimum motion level across the subject timeseries are targeted for removal. Real motion should correspond with an interruption in the grayordinate plot (indicated by red arrow), which is not the case before the application of a respiratory notch filter for many identified frames (marked by black arrows). After the filter is applied, many frames show a decrease in FD and are no longer targeted for frame removal. However, high motion frames caused by spontaneous subject motion and that correspond to an interruption in the grayordinate plot are still targeted for frame removal.

Conventional motion censoring techniques remove high motion frames above a given threshold in an effort to mitigate BOLD signal disruptions induced by motion. BOLD signal disruptions can be visualized as vertical lines in ‘gray plots’ such as Fig. 2 (Power et al., 2014). Plotted along with the motion trace, these signal disruptions align with high motion frames as described in Power et al. (2014). However, in Fig. 2a, there are also many frames that cross the typical FD threshold of 0.2 mm that do not result in a BOLD signal disruption. This is where the factitious respiratory artifact described in Fair et al., is evident. As previously reported by Fair and colleagues, the artifact can be removed by applying a notch filter at the frequency of respiration to the movement trace (Fair et al., 2020). Filtered head movement data shown in Fig. 2b demonstrates that frames with high FD due to spontaneous head motion continue to be censored using a typical threshold of 0.2 mm (Power et al., 2013), whereas frames with motion due to respiration alone are now retained. See SI “Motion Artifact Reduction Analyses” and SI Fig. 2 for additional validation of spontaneous head motion artifact reduction.

### 3.3 Filtering Increases data retention and improves fc estimates

**Fig. 3:**
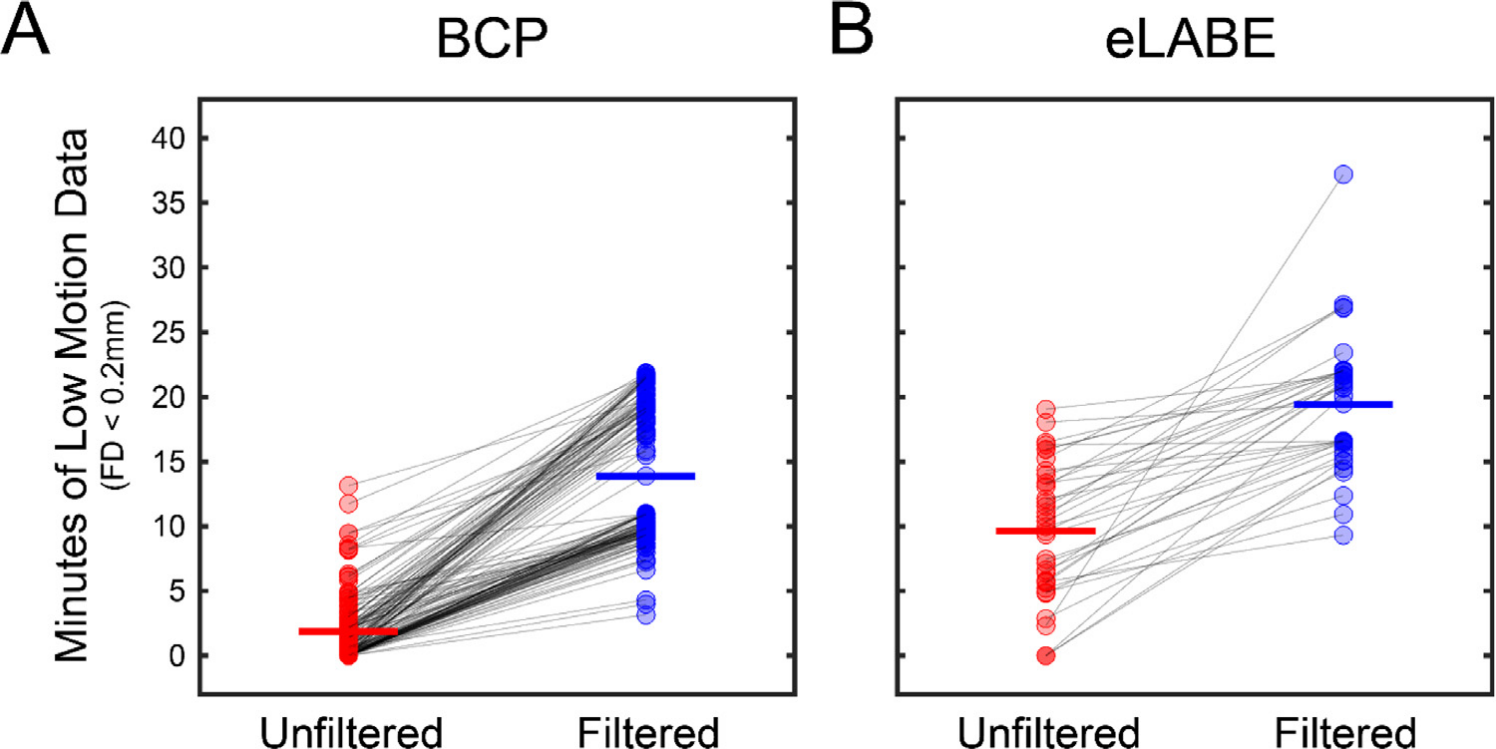
**Linked line representation of data retention by cohort.** Minutes of usable data before and after application of a notch filter applied in the range of respiration in the (A) BCP and (B) eLABE cohorts. In both cohorts, application of the filter greatly increases the average usable minutes of data (from 1.8 ± 2.5 to 13.9 ± 5.2 in the BCP cohort and from 9.6 ± 5.3 to 19.4 ± 5.2 in the eLABE cohort).

Shown in Fig. 3, implementing filtered FD resulted in a substantial increase in the amount of usable (i.e., ‘low motion’, <0.2 mm FD) data retained (from 1.8 ± 2.5 to 13.9 ± 5.2 min in the BCP cohort and from 9.6 ± 5.3 to 19.4 ± 5.2 min in the eLABE cohort).

**Fig. 4:**
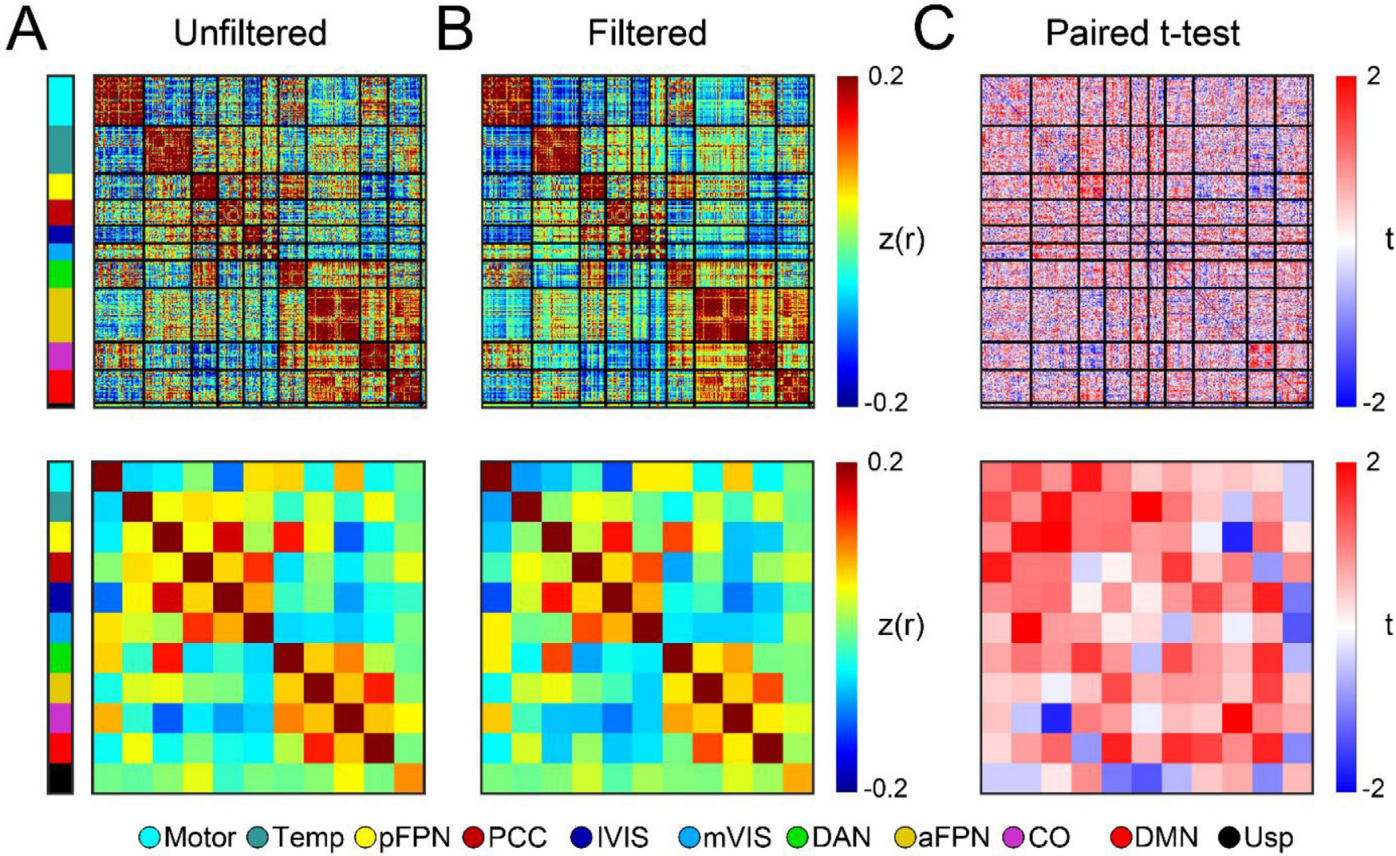
**Functional connectivity within and between brain networks.** (A) Functional connectivity strength across defined cortical parcellations (top row) and their network averages (bottom row) is demonstrated in the BCP cohort for unfiltered data. (B) Functional connectivity matrices in the same BCP subjects after application of the notch respiratory filter across identical functional networks. (C) Statistical analysis using a paired *t*-test comparing the two approaches (unfiltered vs filtered) across functional networks. Here, stronger red indicates higher magnitude fc estimates when filtering data. Networks include: motor, temporal lobe (Temp), posterior frontoparietal (pFPN), posterior cingulate cortex (PCC), lateral visual (lVIS), medial visual (mVIS), dorsal attention (DAN), anterior frontoparietal (aFPN), cingulo-opercular (CO), default mode (DMN), and unassigned (Usp).

Further, for each individual participant, increasing the number of usable frames results in higher magnitude fc measurements, and the parcels that make up a given RSN have more uniform connectivity structure within and between RSNs. This is depicted in Fig. 4, where we see stronger, less noisy within and between RSN connectivity when going from unfiltered (Fig. 4a) to filtered FD (Fig. 4b). The differences in fc estimates that occur and their directionality are represented by the t-statistic in the regions identified in Fig. 4c when using the conventional FD cutoff of 0.2 mm. Stronger red cells indicate higher magnitude fc estimates when filtering data.

**Fig. 5:**
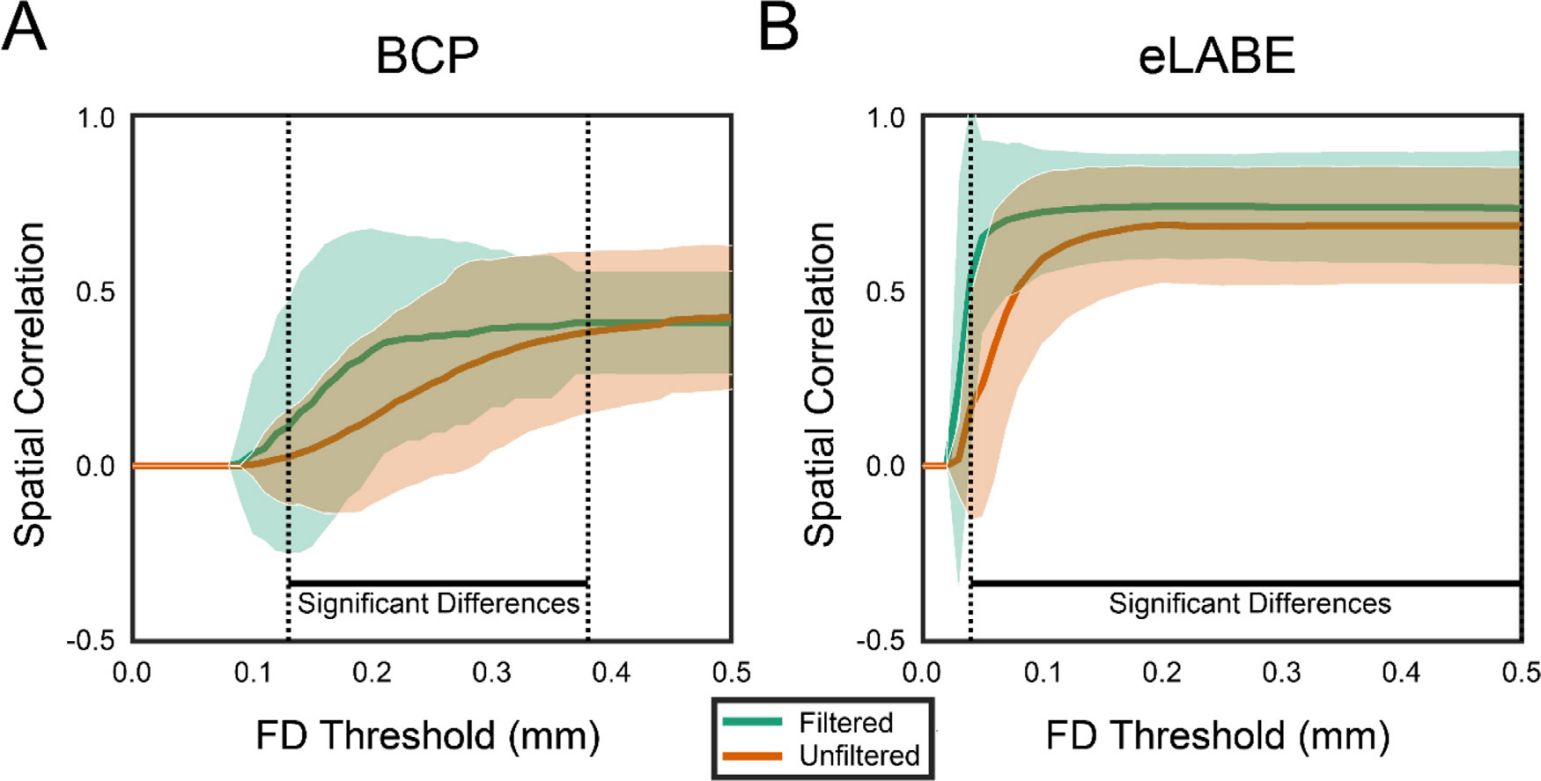
**Reliability of within subject connectivity across FD thresholds.** Demonstration of mean spatial correlation for FC values using a split-half protocol (shaded regions represent ∼2 standard deviations from the mean). This approach evenly split individual subject data into two groups. A corresponding connectivity matrix was created for each group, with the spatial correlation across various FD thresholds then computed for both unfiltered and filtered data. Greater spatial correlation is observed across all FD thresholds for the filtered timeseries, indicating greater reliability of functional connectivity.

In addition to stronger connectivity values, Fig. 5 shows that filtering the FD trace increases the reliability of estimated fc values. The plot depicts the mean correlation between split-half connectivity matrices generated with both filtered and unfiltered FD. In these results, filtered FD data converges to higher correlation values for lower FD thresholds indicating increased reliability. The reliability curve for the eLABE cohort likely converges to a higher correlation value than the BCP cohort since the eLABE cohort contains more usable data in each matrix.

### 3.4 Notch filter cutoffs differ by age

**Fig. 6:**
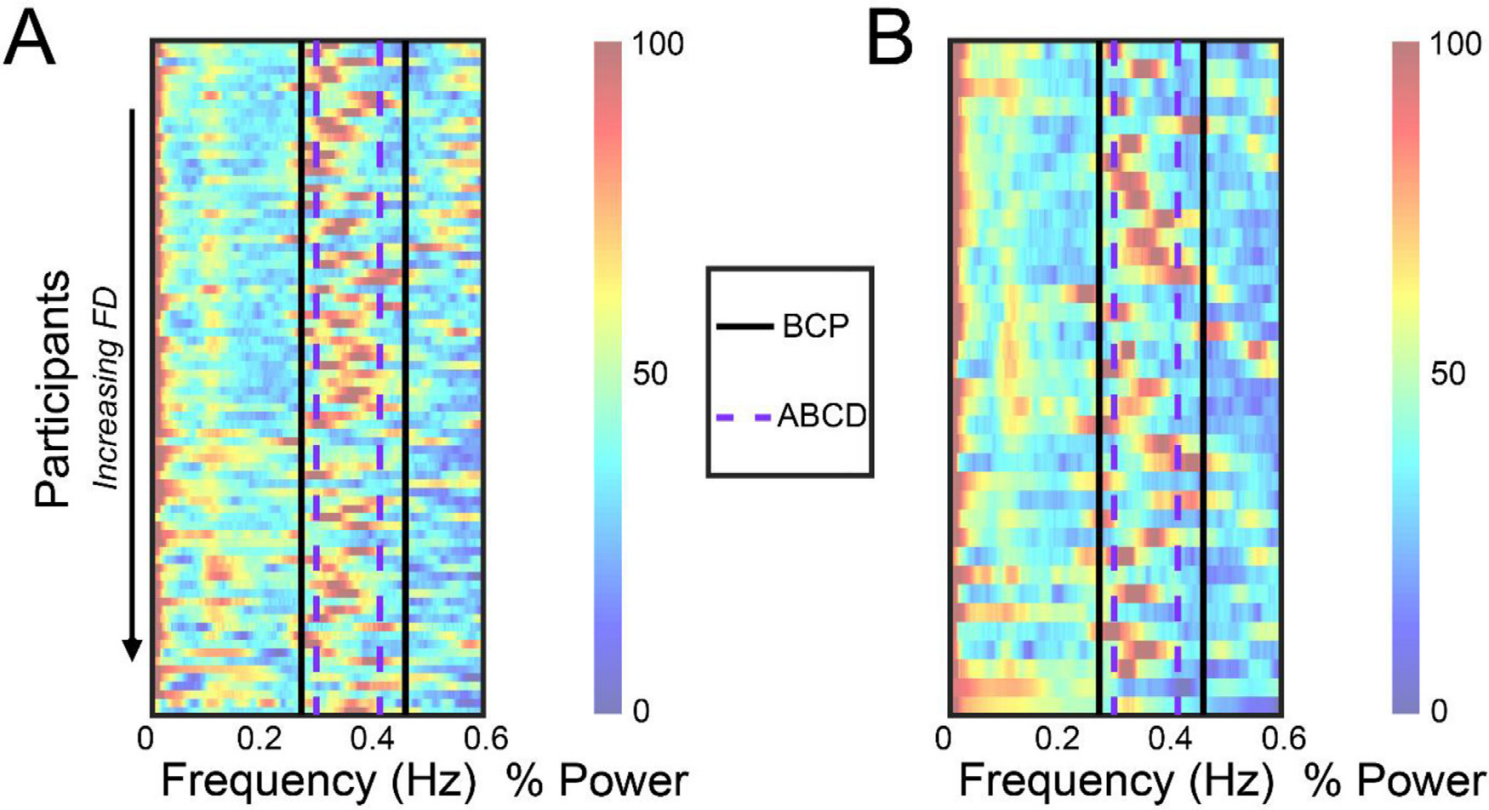
**Frequency domain representations of multiband rs-fMRI timeseries data with notch frequency cutoffs.** Power spectra representation in the Y-direction of the (A) BCP cohort and (B) eLABE cohort organized by FD with lowest motion subjects at the top. The red band between 0.25 and 0.5 Hz indicates respiratory-related artifact. Filter cutoffs suggested by Fair et al., based on the ABCD cohort are marked in dashed purple. This range does not fully encapsulate the respiratory band for all subjects in either cohort, with the red band often extending beyond the ABCD filter cutoff. The cutoff generated from the BCP data is marked in black, offering a more suitable range that captures the respiratory band for most subjects. In order to most effectively remove the variable respiratory artifact in this age group, a slightly wider filter of 0.25 to 0.5 Hz should be applied for subjects age 8–24 months.

Initially, the ABCD notch filter cutoffs from Fair et al., were applied to the toddler data. However, after examining the power spectra in Fig. 6, it was noted that the ABCD cutoffs did not fully encapsulate the spike in signal power caused by respiration in the toddlers. This is because there are higher, more variable respiratory rates at this age, and thus different filtering parameters would better suit this cohort.

**Fig. 7:**
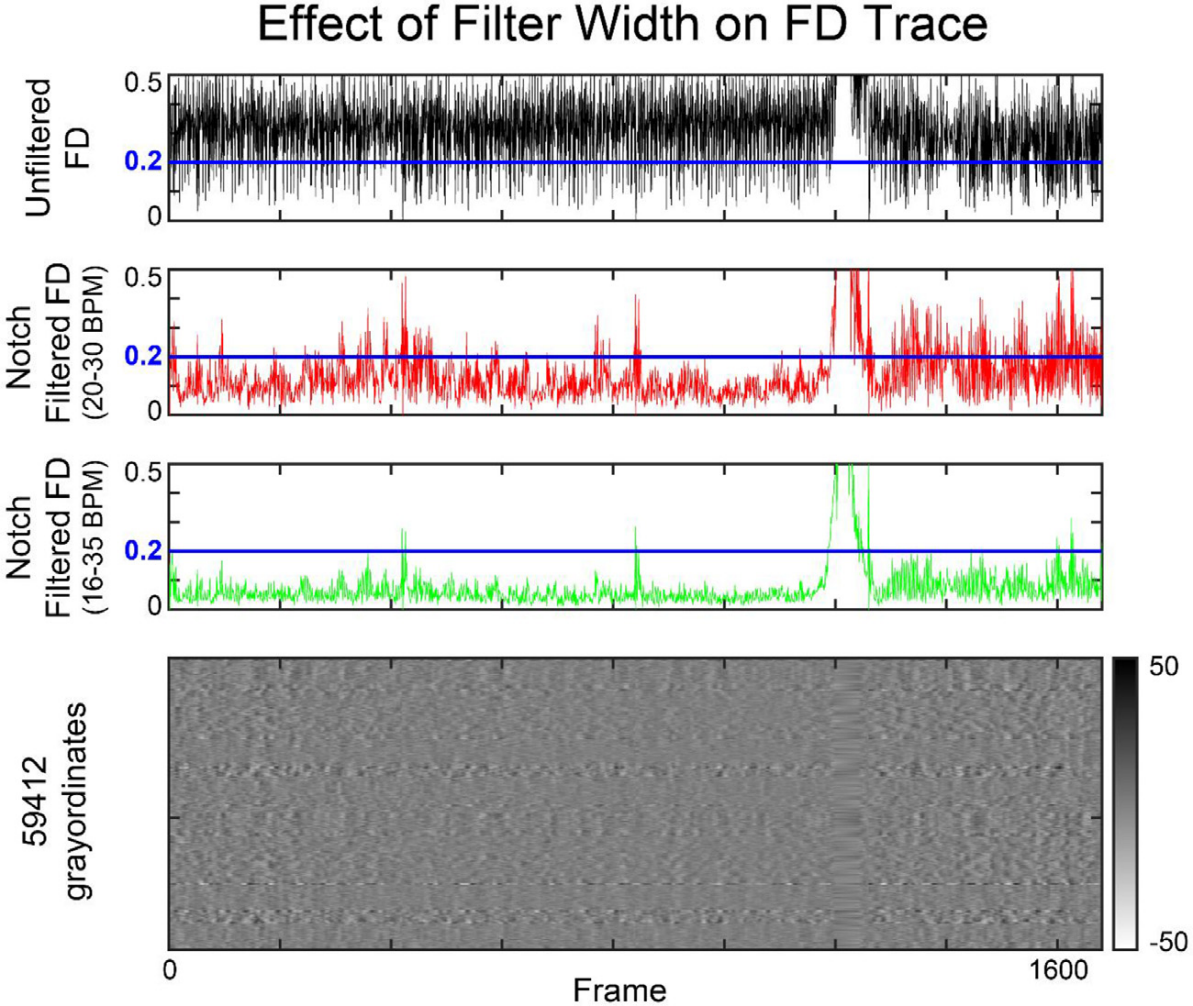
**FD trace resulting from application of various notch filters.** The FD trace for a single subject is presented with various notch filter cutoffs represented. The tested ranges offer the maximum and minimum values of the high power band for BCP and eLABE subjects in the Y-direction indicating respiratory artifact. Across all tested filters, application reduces the power of FD as seen by a downward shift in the mean FD value. The filter with a wider cutoff range of 16–35 bpm (lime) shows a more dramatic FD shift (compared to the narrower filter presented in red from 20 to 30 bpm), with much smaller FD oscillations in the resulting trace. However, across all ranges, FD spikes indicative of spontaneous motion still surpass the FD threshold and would be targeted for frame censoring.

Subsequently, data-driven cutoffs, defined as the second and third quartile frequency peaks of the BCP power spectra, were determined to be 0.28 and 0.48 Hz, and were applied to data from both cohorts. Shown in Fig. 6b, there were several subjects in the eLABE dataset that had lower respiratory rates, and as a result these participants would lose a significant number of frames due to respiratory motion when applying the more narrow BCP filter. In order to fully capture the variability in respiratory rates of this age range, the optimal filter cutoffs for the entire age group were marginally extended to 0.25 and 0.50 Hz. When the slightly wider filter is applied, the respiratory artifact is removed, thereby increasing the number of frames retained. Further still, choosing a wider than necessary filter results in power loss in the overall trace, as seen in Fig. 7, where the overall signal is shifted to lower FD values as the width of the notch filter increases.

## 4 Discussion

In this work, we have shown that respiratory-driven motion artifact is present in MB BOLD data of populations as young as 8 months of age and must be appropriately addressed when processing fMRI data in this age range. Critically, we have shown that filtering of FD traces to remove this artificial head motion can be successfully and effectively implemented in infants and toddlers. Further, we have demonstrated these efforts are most successful when using an age-specific filter design that appropriately captures differences in respiratory rate. Successful application of this filtering approach results in both increased data retention and improved data quality including higher magnitude fc measures, illustrating its critical importance in investigations utilizing this modality in this age group.

### 4.1 Motion in adult and toddler rs-fMRI data

In adult rs-fMRI investigations, spontaneous motion during data collection has been shown to bias connection strength based upon physical distance, introducing colored noise into measured results (Power et al., 2012; Satterthwaite et al., 2012; Van Dijk et al., 2012). One common way to address this has been by implementing frame censoring (i.e., “scrubbing”) in rs-fMRI post-processing. In scrubbing, head motion estimates are used to identify frames with spontaneous motion greater than a designated FD threshold (typically ∼0.2 mm), which are then removed from the dataset. However, important recent work using both adult and adolescent data has additionally shown head motion caused by respiratory efforts manifests in the tracings used to identify motion-corrupted frames in MB BOLD data. However, unlike spontaneous head motion, this respiratory-driven head motion does not bias fMRI correlation strength between regions based upon anatomic distance, and thus should not be incorporated when identifying frames for motion censoring (Fair et al., 2020). Thus, traditional censoring approaches which conflate respiratory motion with spontaneous motion in the FD traces may result in unnecessary censoring of large quantities of data thereby reducing the power of fc analyses. This has led to the recent advent of frequency-based filtering approaches which successfully identify frames for removal due to spurious head motion only (Fair et al., 2020). This is critical as it has been shown that increased amounts of rs-fMRI data result in more reliable measures of connectivity (Gordon et al., 2017; Laumann et al., 2015).

It was previously assumed that this respiratory motion artifact was not present in toddler rs-fMRI data given that their smaller chest sizes and faster breathing rates were thought to have minimal effects on the B0 field (Power et al., 2019). Moreover, prior studies of toddlers primarily utilized single band rs-fMRI sequences (Eggebrecht et al., 2017; Smyser et al., 2016; Toulmin et al., 2015; Doria et al., 2010; Smyser et al., 2010; Gao et al., 2009; Fransson et al., 2007), which were shown in Fair et al., to be impacted by respiration to a lesser extent than MB data due to the lower in sampling rate. Subsequently, investigation for the presence and severity of this artifact in this population has not been previously undertaken. However, herein we have shown across two independent cohorts that, similar to adult data, head motion due to respiration is indeed prevalent in FD traces and significantly alters frame censoring results in infants and toddlers. While the artifact is most pervasive in the phase encoding direction, it can “leak” into other planes by means discussed in Fair et al., and mix with true head motion in parameter estimates. This makes it difficult to disentangle real head motion from artifact in the non-phase encoding planes, so filtering must be applied in all directions. Since there are multiple ways in which respiratory artifact can corrupt BOLD data, further work is needed to separate individual components. Critically, accurately identifying and correcting for this artifact using frequency-specific filters during frame censoring leads to significant increases in data retention and the reliability and strength of fc measures that are comparable to results in older populations (Fair et al., 2020; Gratton et al., 2020). This is of particular importance given that large amplitude, spontaneous movements are relatively common in sleeping toddlers and multiple scanning sessions are often impractical, factors which can make acquiring large quantities of rs-fMRI data challenging and frame retention increasingly important in these analyses.

### 4.2 Filter Selection to remove respiratory artifact

There are several filter types that can achieve removal of respiratory artifact, including bandpass, low pass, and notch filters. Both bandpass and low pass filters decrease the overall power of the FD trace, which shifts the entire trace to lower FD values. This is something to be cognizant of with frame censoring, as the conventional 0.2 mm threshold for the identification of low motion frames may no longer be applicable. In order to keep with established convention and to filter out only a narrowly defined frequency range, a notch filter is likely most suitable. When selecting notch filter cutoffs, it is important to consider the width of the filter. Similar to bandpass and low pass filters, increasing the width of the notch too far may also decrease the power of the trace. Therefore, it is important to limit the width of the notch cutoffs to maintain applicability of conventional scrubbing thresholds. For 8 to 24 months of age, we recommend using a slightly wider cutoff of 0.25 to 0.5 Hz in order to fully capture the spectrum of respiratory rates for this particular age group without substantial power loss. Though in principal the cutoff frequencies could be calculated on the individual level, prior work has shown doing so is challenging in practice and provides no additional advantage over the group-level estimates (Fair et al., 2020).

### 4.3 Necessity of age-specific rs-fMRI acquisition and analysis methods

Application of rs-fMRI in infants and toddlers has provided unique insights into the functional architecture of the developing brain and enabled characterization of both normal and disordered brain development (Eyre et al., 2021; Azhari et al., 2020; Graham et al., 2015; Zhang et al., 2019; Eggebrecht et al., 2017; Marrus et al., 2018; Gao et al., 2009; Fransson et al., 2007; Lin et al., 2008). However, when determining optimal data acquisition and processing approaches for use in these age group, special attention must be paid to the unique characteristics of this target population. Principle among these are differences across key variables including motion patterns during data collection, head size, and tissue contrasts. There have now been numerous strategies developed to specifically address the unique challenges inherent to studying this age group (Kim et al., 2013; Zhang et al., 2016; Hazlett et al., 2017). For example, age-specific techniques to minimize subject motion during data collection (i.e., collecting sleep scans with immobilization) have been developed and applied (Howell et al., 2019). Further, age-specific atlases which address differences in head size, tissue contrast properties, and relative growth patterns have been created to facilitate successful image registration procedures. In this investigation, we demonstrate that age-specific methods for identifying, characterizing, and removing the effects of subject motion during rs-fMRI data processing, including those driven by respiratory artifact, are another critical element of these procedures.

### 4.4 FIRMM for real-time head motion estimation

**Fig. 8:**
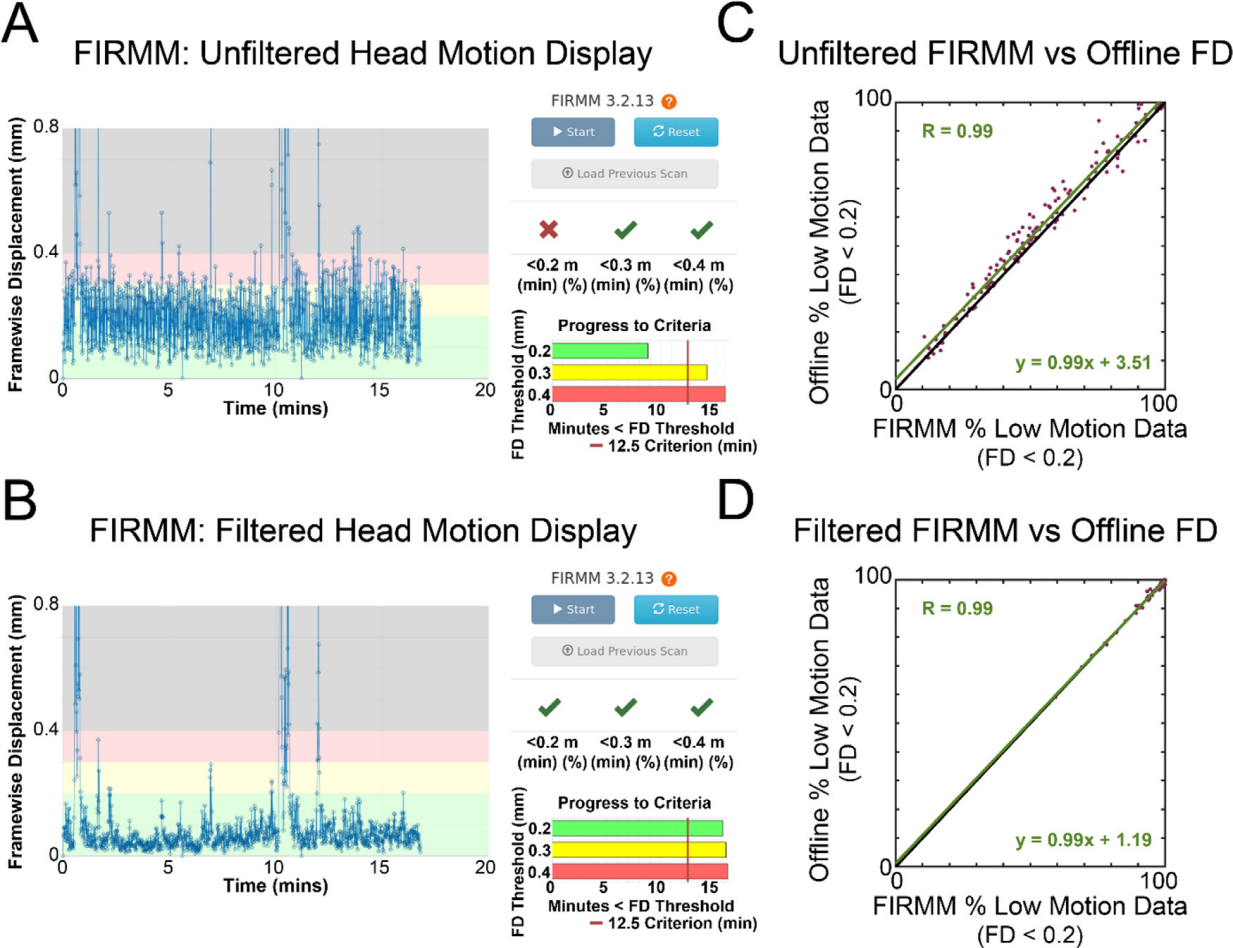
**Comparison of on-line (FIRMM) and off-line (cross_realign3d_4dfp) motion estimation.** The FD trace computed scanner-side for a representative eLABE subject (A) without filtering and (B) with filtering. Percentage of usable frames for all eLABE participants determined using on-line calculated FD estimates compared to off-line calculated FD estimates using both (C) unfiltered and (D) filtered data.

Given the challenges of acquiring low motion rs-fMRI data in younger populations (Turesky et al., 2021; Raschle et al., 2012), it may be highly valuable to obtain an accurate assessment of data quality during the scan. The results shown here, including the use of population specific respiratory and head circumference settings, can be readily implemented using FIRMM (Framewise Integrated Real-Time MRI Monitoring) (Dosenbach et al., 2017), a scanner-side software platform that provides real-time head motion estimates throughout the entirety of the study for both unfiltered and filtered FD. These estimates match closely with what is estimated in post-scanner processing, shown for a representative eLABE subject in Fig. 8.

### 4.5 Limitations

When comparing across the BCP and eLABE cohorts, there were some collection differences that could not be corrected for post-acquisition. Namely, some BCP rs-fMRI acquisitions were collected with a TR of 720 ms, while the majority were collected with a TR of 800 ms. Despite these differences, the respiratory artifact high power band was observed across all TR ranges (SI Fig. 1), indicating that this phenomenon may extend beyond only cohorts with specific acquisition protocols. Further, as shown in SI Fig. 1, application of the respiratory filter effectively removes respiratory artifact in each TR group independently. In addition, application of the respiratory filter was completed using two different functions in MATLAB (iirnotch and butter) based upon institutional availability of software packages. Again, there was no observable difference between these two approaches, demonstrating flexibility when applying the suggested filter.

This work was completed using data collected from two healthy infant and toddler cohorts; therefore, best practices for application of these methods in non-normative population studies requires further investigation. Additionally, this work was limited to children down to 8 months of age. Further investigation into even younger populations also remains necessary due to the higher sampling rate required to avoid aliasing into lower frequencies caused by an increase in respiratory rate.

## 5 Conclusion

This work has shown that apparent head motion due to respiration is present in rs-fMRI data in infants and toddlers. Critically, this artificial head motion spuriously decreases the amount of usable (i.e., low motion) rs-fMRI data. Applying an age-specific notch filter to the FD trace can readily and effectively remove this artifact, thereby optimizing frame retention and increasing fc measure reliability. Critically, these approaches can be readily and successfully applied in younger populations using existing software applications both in real-time at the scanner during data collection and in off-line post-processing. When working with data from younger populations, it is important to adjust notch filter cutoffs appropriately for the population being studied in a data-driven manner. Successful application of these approaches is necessary for both improving understanding of early functional brain development and defining brain-behavior relationships during this critical developmental window.

## CRediT statement

**S. Kaplan:** Writing – original draft, Writing – reviewing & editing, Formal Analysis, Visualization. **D. Meyer:** Writing – original draft, Formal analysis, Visualization. **O. Miranda-Dominguez:** Methodology, Formal analysis. **A. Perrone:** Methodology, Formal analysis. **E. Earl:** Methodology, Formal analysis. **D. Alexopoulos:** Formal analysis. **D.M. Barch:** Funding acquisition, Investigation. **T.K.M. Day:** Investigation. **J. Dust:** Formal analysis. **A.T. Eggebrecht:** Investigation. **E. Feczko:** Investigation. **O. Kardan:** Investigation. **J.K. Kenley:** Formal analysis. **C.E. Rogers:** Funding acquisition, Investigation. **M.D. Wheelock:** Formal analysis, Writing – reviewing & editing. **E. Yacoub:** Investigation. **M. Rosenberg:** Funding acquisition, Investigation. **J.T. Elison:** Funding acquisition, Investigation. **D.A. Fair:** Conceptualization, Funding acquisition, Investigation. **C.D. Smyser:** Writing – original draft, Writing – reviewing & editing, Funding acquisition, Investigation, Supervision.

## Data and code availability statement

All data and code developed and/or used specifically for this study can be made available to qualified investigators by written request through the study authors under the guidance of a formal data sharing agreement between institutions that includes: 1) using the data only for research purposes and not attempting to identify any participant; 2) limiting analyses to those described in both institutions IRB-approved protocols; and 3) no redistribution of any shared data without a data sharing agreement.

## Declaration of Competing Interest

Oregon Health and Sciences University and Eric Earl have a financial interest in Nous Imaging, Inc., a company that may have a commercial interest in the results of this research and technology. Additionally, Dr. Damien Fair is co-founder and equity holder of Nous Imaging Inc., which has licensed FIRMM Software. These interests have been reviewed and managed by the University of Minnesota and Oregon Health and Sciences University in accordance with their Conflict of Interest policies.

## Appendix Supplementary materials

Image, application 1
