# Supplementary material for: Filtering respiratory motion artifact from resting state fMRI data in infant and toddler populations
Source: Neuroimage. 2022 Feb 15;247:118838. doi: 10.1016/j.neuroimage.2021.118838 (PMC8803544; doi:10.1016/j.neuroimage.2021.118838)
Supplement: Supplementary file 1 [file mmc1.docx]

**Supplemental Information**

**Baby Connectome Project (BCP) Repetition Time (TR) Replication**

To verify that the filtering protocol is applicable for both TRs in the BCP data, the power spectra and resultant connectivity matrices were computed on each TR group independently and are depicted SI Fig. 1. Note the similar respiratory frequency bands and effect of filtering on connectivity estimates for data acquired using both TRs.


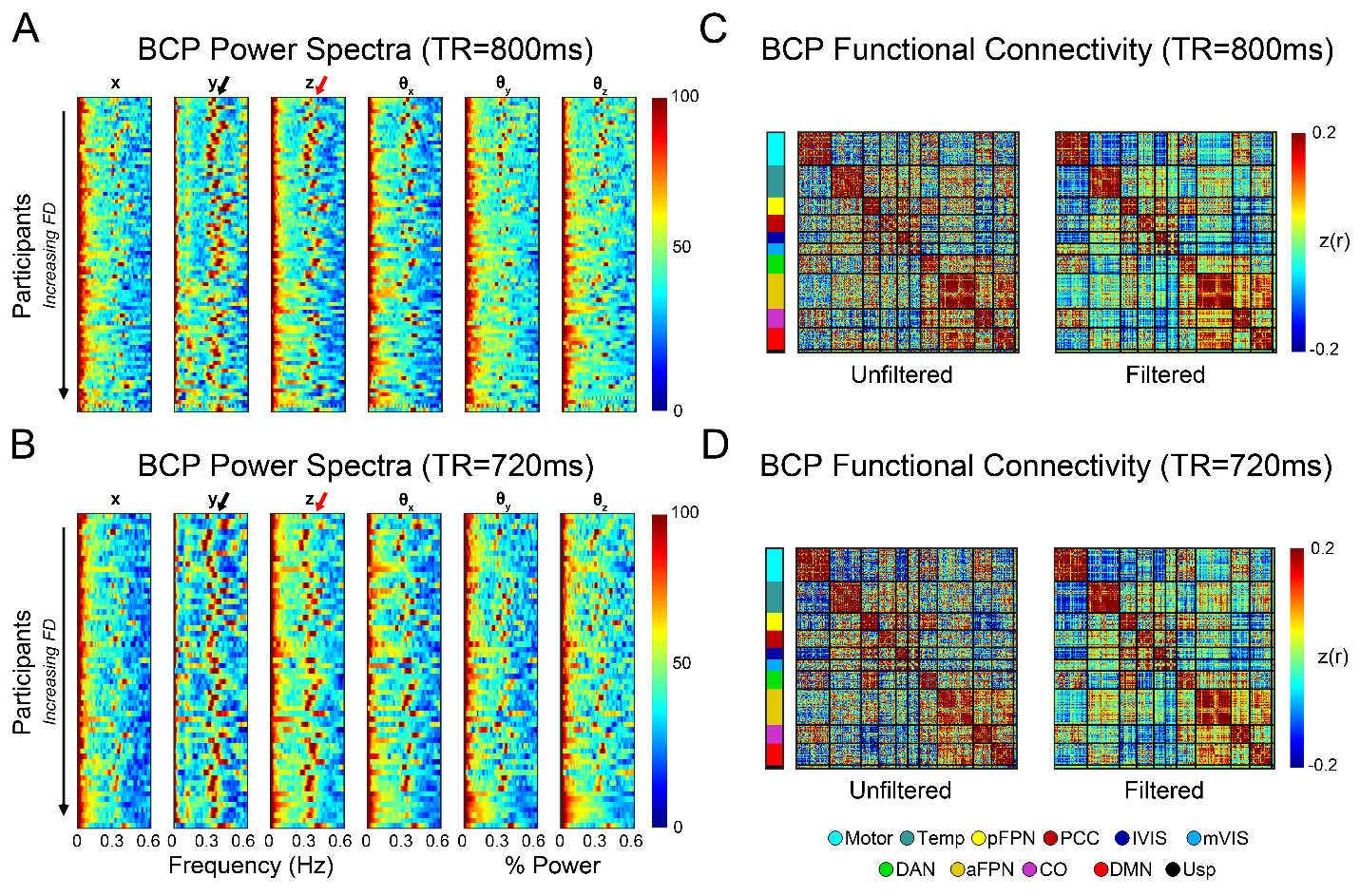


**Supplementary Figure 1: Application of filtered FD to different TRs.** The power spectra for BCP subjects scanned with TR of (A) 800ms and (B) 720ms. Subjects are ordered by mean FD, with the lowest motion subjects organized at the top. Red and black arrows indicate power spike due to respiration. Filtering the head motion estimates for nuisance regression and FD computation results in increased and less noisy connectivity estimates for subjects with a TR of both (C) 800ms and (D) 720ms.

**Motion Artifact Reduction Analyses**

To assess the extent to which various frame censoring techniques reduce general motion artifact, we computed the QC-FC relationships with distance (Ciric et al., 2017) for three censoring methods: including frames less than 0.2 unfiltered FD, frames less than 0.5 unfiltered FD, and frames less than 0.2 filtered FD. Results for all three censoring methods are shown in SI Fig. 2. While all three methods show minimal distance dependence, compared to the conventional method of censoring at 0.2 unfiltered FD, both raising the unfiltered FD threshold to 0.5 and filtering FD increase the amount of usable data by about 12 minutes on average.


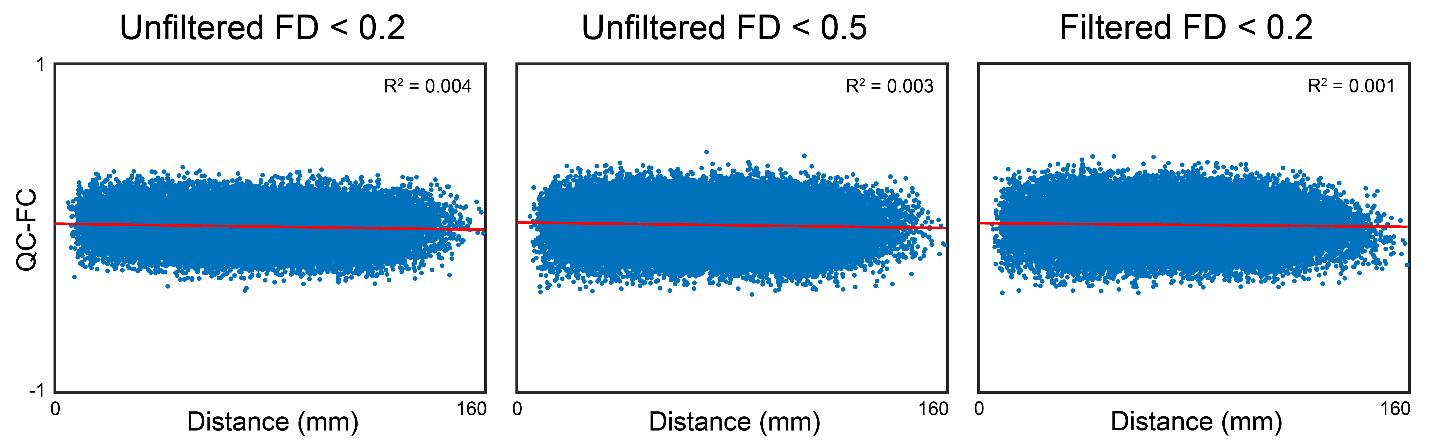


**Supplementary Figure 2: Distance dependence of FC values related to motion.** The correlation between FC values and motion are plotted on the y-axis against the distance between parcel pairs on the x-axis for censoring at (left) 0.2 unfiltered FD, (center) 0.5 unfiltered FD, and (right) 0.2 filtered FD. Linear trends are depicted as red lines.

Importantly, as indicated by red arrows in SI Fig. 3a, raising the FD threshold and using an unfiltered tracing allows large-amplitude motion to corrupt FC estimates, whereas using filtered FD ensures that these motion-corrupted frames are removed from analyses. Correspondingly, as shown in SI Fig 3b, fc estimates generated using filtered FD are both increased in magnitude and less noisy than those produced using a higher unfiltered FD threshold.


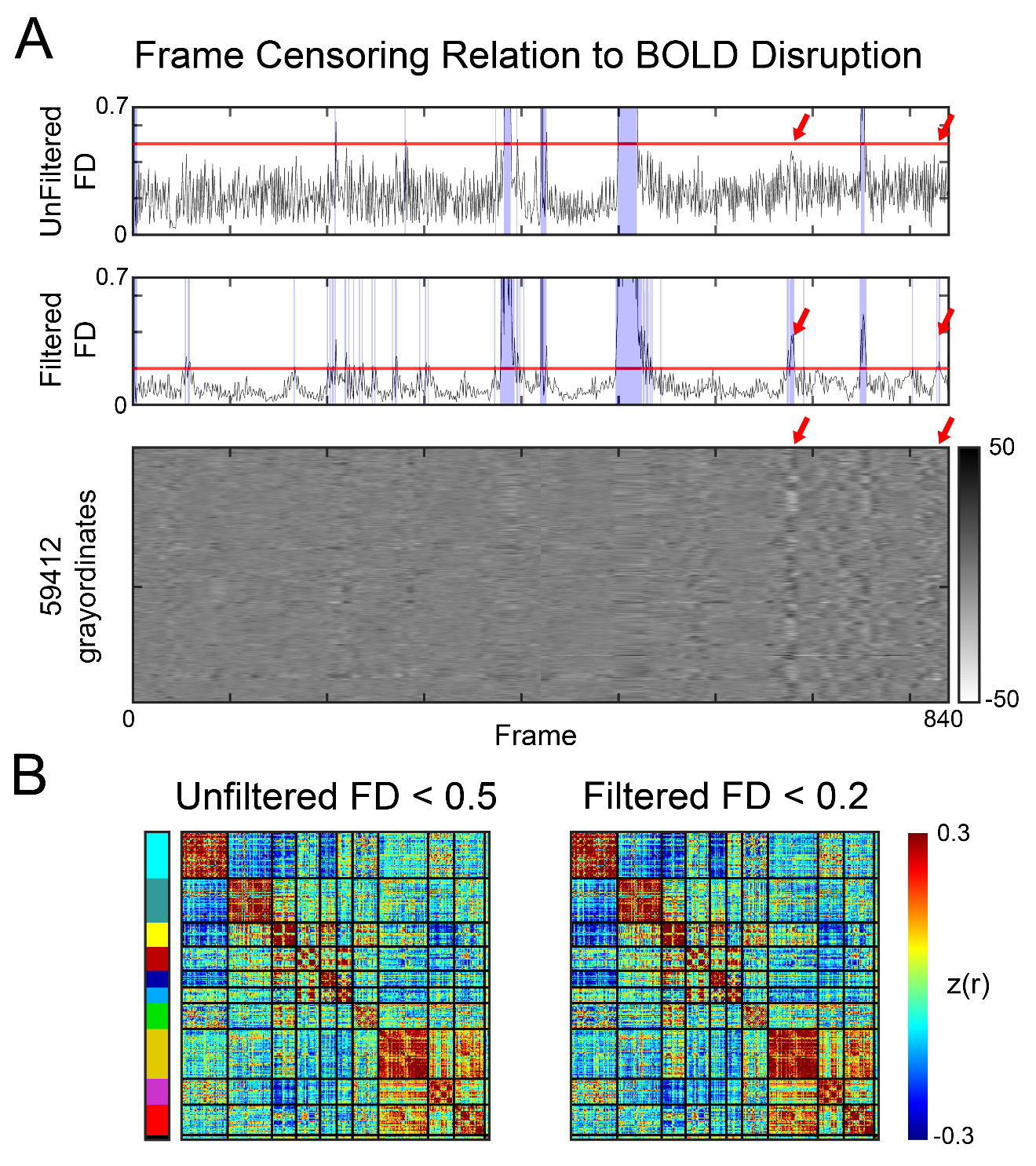


**Supplementary Figure 3: BOLD Signal Disruption Comparison.** (A) Unfiltered and filtered FD traces and the corresponding grayordinate plot across time for a representative subject. Red horizontal lines delineate FD thresholds (0.5 for unfiltered and 0.2 for filtered), and blue shading indicates censored frames. Representative examples of frames that correspond to BOLD signal disruption that are censored using filtered FD but not using unfiltered FD at higher thresholds are indicated by red arrows. (B) Corresponding connectivity matrices for each filtering approach.
